# Prolonging calcineurin inhibitor therapy post kidney allograft failure: a prospective study

**DOI:** 10.1080/0886022X.2025.2483386

**Published:** 2025-03-30

**Authors:** Rita Leal, Pedro Fragoso, João Venda, José Gomes, Maria Inácio, Maria Guedes Marques, Luís Rodrigues, Lídia Santos, Catarina Romãozinho, Francisco Caramelo, Helena Oliveira Sá, António Martinho, Arnaldo Figueiredo, Rui Alves

**Affiliations:** a Nephrology Department, ULS-Coimbra, Coimbra, Portugal; b Faculty of Medicine, University of Coimbra, Coimbra, Portugal; c Centro de Histocompatibilidade do Centro, Instituto Português do Sangue e Transplantação, Coimbra, Portugal; d Urology and Kidney Transplantation Unit, ULS-Coimbra, Coimbra, Portugal

**Keywords:** Allosensitization, epitope matching, graft nephrectomy, immunosuppression withdrawal, kidney graft failure, retransplantation

## Abstract

**Background:**

The optimal immunosuppressive (IS) withdrawal strategy after kidney allograft failure remains unclear. This study evaluated the effects of prolonged calcineurin inhibitor (CNI) therapy on HLA sensitization, graft intolerance syndrome (GIS), and key clinical outcomes.

**Methods:**

We conducted a prospective cohort study involving 90 adult patients with kidney allograft failure who were candidates for re-transplantation. Patients were divided into two groups: Rapid withdrawal group (discontinuation of all IS except low-dose prednisolone) and Prolonged CNI Group (maintenance of CNI for six months plus low-dose prednisolone). Outcomes assessed over a 12-month follow-up period included HLA sensitization, defined as an increase in calculated panel reactive antibody (cPRA) and the development of de novo donor-specific antibodies (dnDSA), GIS incidence, re-transplantation, hospitalization rates, and mortality.

**Results:**

No significant differences were observed between the groups regarding HLA sensitization one-year postgraft failure. A composite outcome of cPRA increase, dnDSA, and GIS did not differ between the groups. When evaluated separately, GIS occurred less frequently in the Prolonged CNI Group (4.8% vs. 23%; *p* = 0.015). Patients who continued CNI maintained better residual kidney function at 6 months (800 vs. 200 mL, *p* = 0.001) and experienced lower all-cause hospitalization rates (12% vs. 30%, *p* = 0.036), with comparable retransplantation and mortality rates. Graft removal and higher HLA mismatches were independently linked to increased sensitization at 12 months.

**Conclusions:**

Prolonged CNI therapy for six months postallograft loss did not prevent HLA sensitization but reduced the incidence of GIS and preserved residual kidney function without increasing hospitalization or mortality.

## Introduction

The number of kidney transplant (KT) patients returning to dialysis after graft failure is increasing, making it the fourth leading cause of dialysis initiation [1,2]. Retransplantation offers the most significant survival benefit for eligible patients, even for those in challenging subgroups such as diabetics or the elderly [3,4]. Unfortunately, access to a subsequent KT is often compromised by HLA sensitization, with patients waiting for retransplantation accounting for most of the highly sensitized individuals on the waitlist [5,6].

Advances in immunogenetics have enhanced our understanding of the dynamics of allosensitization and emphasized strategies to reduce the number of highly sensitized patients awaiting a subsequent KT [7,8]. Improved HLA matching of the first allograft, now achievable at the molecular level, preventing cumulative sensitizing events, and ensuring better adherence to immunosuppression (IS) therapy are well-known methods for decreasing sensitization rates and improving graft survival [9–11].

However, after allograft loss, unresolved issues, such as managing IS withdrawal, may also influence sensitization [12–14]. Tapering off IS for patients returning to dialysis is complex and lacks strong evidence or universally accepted recommendations, resulting in significant variability in clinical practice. A Portuguese survey revealed substantial discrepancies in how nephrologists manage IS withdrawal after graft loss [15], a trend also noted in the United States and South Asia [16,17]. Continuing IS after allograft loss may lower the risk of graft intolerance syndrome (GIS), preserve residual kidney function (RKF), and reduce sensitization rates. However, these potential benefits must be balanced against the heightened risk of infection, cardiovascular disease, and malignancy.

Although previous studies have examined the role of continuing or gradually tapering IS therapy in preventing allosensitization, many have limitations, such as being retrospective, having small sample sizes, or involving heterogeneous groups, leading to inconsistent results [18–27].

We aimed to prospectively assess the impact of prolonging calcineurin inhibitors (CNI) after graft loss on HLA sensitization, GIS, hospitalization rates, and mortality and identify risk factors for developing sensitization one year after allograft loss.

## Methods

### Study population

**Figure 1.**
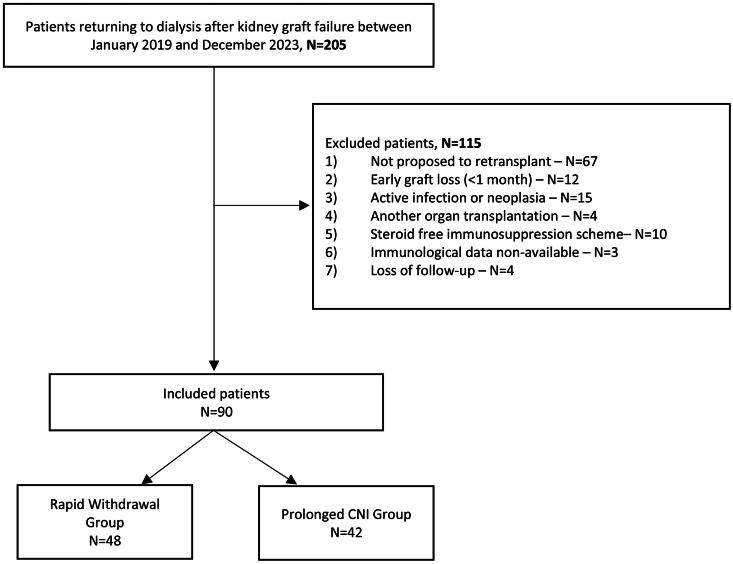
Flowchart of patients included in the study.

We prospectively reviewed all patients with death-censored kidney allograft failure who started maintenance dialysis between January 2019 and March 2023 at Centro Hospitalar e Universitário de Coimbra - ULS Coimbra, a tertiary university hospital that monitors approximately 1,600 KT recipients under the care of a team of 13 nephrologists. Adult patients eligible for re-transplantation were included in this study. The exclusion criteria are presented in Figure 1. The follow-up period ended in March 2024 or at the time of retransplantation or death, with a mean follow-up period of 35 ± 13 months. Written informed consent was obtained from all the patients. The study protocol was approved by the Ethical Committee of Centro Hospitalar e Universitário de Coimbra and Faculdade de Medicina da Universidade de Coimbra (#CE-002-2020). It was conducted following the Declaration of Helsinki.

### Group definition

At our center, following graft failure, all patients discontinue antiproliferative drugs and mammalian target of rapamycin (mTOR) inhibitors within the first few days. In 2019, following the approval of the study protocol, a structured approach to CNI withdrawal was introduced, standardizing the continuation of CNI for 6 months postgraft failure. Given the absence of formal guidelines, patients were prospectively assigned to either prolonged CNI or rapid withdrawal based on the participating nephrologist’s individual practice experience and preference. This allocation was independent of patient clinical features, reflecting a quasi-randomization design. This methodology enabled the establishment of two distinct groups:

- Rapid Withdrawal Group: Immediate withdrawal of CNI/mTOR inhibitor therapy and continuation of prednisolone 5 mg daily for at least one year.
- Prolonged CNI Group: maintenance of CNI for 6 months and prednisolone 5 mg daily for at least 1 year.

### Data collection and outcomes

Data on demographics, clinical characteristics, and IS withdrawal were collected at the start of dialysis (T0). Study visits occurred at 6 months (T6 − 6 ± 1.5 months) and 12 months (T12 − 12 ± 3.2 months) after allograft loss and then annually until the end of the follow-up. At each visit, patients were assessed for all-cause hospitalization, sensitization events, and GIS, defined as the presence of at least two of the following: fever without documented infection, hematuria, anemia, and malaise. At T6, residual diuresis was measured using a 24-h urine collection, and CNI trough levels were assessed in the Prolonged IS group. Blood samples were collected at T0, T6, and T12 for histocompatibility and immunogenetic analysis.

The primary outcomes of our study were to assess the impact of prolonged CNI on HLA sensitization and the risk of GIS in the first year following graft failure. HLA sensitization was defined as an increase in calculated panel reactive antibodies (cPRA) and the development of *de novo* donor-specific antibodies (dnDSA). cPRA was evaluated as both a continuous and a categorical variable by defining four sensitization categories: cPRA 0-19%, cPRA 20-84%, cPRA 85-97%, and cPRA ≥ 98% (highly sensitized patients). Highly sensitized patients at T0 were excluded from the increased cPRA categorical outcome at T12. To assess the clinical significance of maintaining CNI, we have also established a composite outcome that included increased cPRA, dnDSA, and GIS at T12 following graft failure. Patients with missing data for at least one of these components were excluded from the analysis, and the endpoint was considered positive if any of the three criteria were met.

As secondary outcomes, we evaluated hospitalization, retransplantation, and mortality. Additionally, we analyzed the risk factors for increased sensitization, which was defined as a composite outcome of cPRA increase and/or dnDSA at T12.

### Histocompatibility and immunogenetic studies

High-resolution HLA typing of the included patients and their paired donors was conducted using next-generation sequencing (NGS) for 11 HLA loci (HLA-A, HLA-B, HLA-C, HLA-DR, HLA-DQ, HLA-DP) using the All Type FAST plex NGS 11 Loci Kit (One Lambda, Canoga Park, CA, USA). The HLA Eplet Registry [28] was used to determine eplet mismatches at the HLA-A, -B, -DRB1, and DQB1 levels. Allelic mismatches were also assessed for HLA-A, HLA-B, HLA-DR, and HLA-DQ loci. Anti-HLA antibodies (class I HLA-A, HLA-B, class II HLA-DR, HLA-DQ) were identified using a single antigen bead assay (Luminex), following the manufacturer’s instructions (One Lambda, Canoga Park, CA, USA), using EDTA-treated sera and analyzed using HLA Fusion 4.6 software. Considering the Portuguese allocation law, a mean fluorescence intensity (MFI) of 1000 was the threshold for antibody positivity to determine unacceptable antigens based on the patient’s allosensitization history. cPRA was determined using HLA allele frequencies from a Portuguese national database of 10.000 potential donors. We have also analyzed our results considering the MFI 2000 cutoff for unacceptable antigens (supplementary material).

### Statistical analysis

Statistical analyses were performed using IBM SPSS Statistics version 27. Categorical variables were described using relative and absolute frequencies, whereas continuous data were reported as mean, median, standard deviation, and interquartile range (25th and 75th percentiles). The T-test or Mann–Whitney test was used to compare the means of normally and non-normally distributed variables. Categorical variables were compared using the χ^2^ test or Fisher’s exact test. A confidence interval of 95% and a p-value of < 0.05 were considered statistically significant. Linear mixed model analysis was used to analyze the relationship between time and cPRA levels, with the IS group as a fixed effect and individual patients as random effects to account for variability. Multivariable logistic regression models were constructed to identify risk factors for cPRA sensitization, including clinically relevant variables and those from the bivariate analysis with a *p*-value <0.05.

## Results

### Demographics and clinical features

Of 205 patients who experienced death-censored graft loss between January 2019 and March 2023, 90 were included in this study **(**Figure 1**)**. Of these, 48 were assigned to the Rapid Withdrawal Group, and 42 were assigned to the Prolonged IS Group. In the Prolonged IS Group, 29 (69%) were on extended-release tacrolimus with a mean dose of 3.08 ± 1.0 mg daily, achieving mean serum levels of 3.07 ± 0.7 ng/dL at 6 months. Thirteen patients (31%) continued on cyclosporine with a mean dose of 77 ± 28 mg/dL and serum levels of 41 ± 18.5 ng/dL at 6 months.

Table 1 summarizes the demographics, clinical features, and histocompatibility data of the entire cohort and the two comparison groups. There were no differences between groups regarding demographics, comorbidities, or chronic kidney disease (CKD) etiology. Induction therapy was predominantly IL-2 monoclonal antibody basiliximab for both groups (*N* = 77, 85.5%). Regarding maintenance IS, 75.6% (*N* = 68) of the patients were under triple IS before KT: 61 patients with antiproliferative plus CNI plus steroids and seven patients under mTOR plus CNI plus steroids. The remaining patients were under double IS with CNI plus steroids. There were no differences in maintenance IS for either group. No significant differences were found in the number of HLA mismatches. All patients had a cPRA < 20% before KT, with 93% (*N* = 84) having a cPRA of 0%. None of the patients had DSAs at the time of transplantation.

**Table 1.** Demographics and clinical features of the total cohort and immunosuppression withdrawal strategy.

	Total *N* = 90	Rapid Withdrawal *N* = 48	Prolonged CNI *N* = 42	*P*
Age at graft loss, years (mean ± sd)	49 ± 11	50.6 ± 10.1	47 ± 10.9	0.11
Female sex, *N* (%)	29 (32.2%)	15 (31.2%)	14 (33.3%)	0.8
Caucasian, *N* (%)	85 (94.4%)	45 (93.8%)	40 (95.2%)	0.75
Diabetes mellitus, *N* (%)	22 (22.2%)	11 (22.9%)	9 (21.4%)	0.86
CKD etiology, *N* (%)				0.24
Glomerular Disease	37 (41.1%)	18 (37.5%)	19 (45.2%)	
Unknown	24 (26.7%)	11 (22.9%)	13 (30.9%)	
Chronic pyelonephritis/ obstructive uropathy	13 (14.4%)	8 (16.7%)	5 (11.9%)	
Genetic	10 (11.1%)	7 (14.6%)	3 (7.1%)	
Diabetic Nephropathy	3 (3.3%)	3 (6.2%)	0	
Vasculopathy	3 (3.3%)	1 (2.1%)	2 (4.8%)	
First graft loss, *N* (%)	81 (90.0%)	44 (91.6%)	37 (88.1%)	0.57
Induction thymoglobulin, *N* (%)	13 (14.4)	7 (14.6%)	6 (14.3%)	0.66
Triple maintenance IS, *N* (%)	68 (75.6%)	36 (75.0%)	32 (76.2%)	0.89
CNI + mTOR + steroids (prednisolone), *N* (%)	7 (7.8%)	5 (10.4%)	2 (4.8%)	0.44
cPRA before KT, median [IQR]	0 [0-15]	0 [0]	0 [0-15]	0.84
DSA before KT, *N* (%)	0 (0%)	0 (0%)	0 (0%)	1
Total mismatch ABDRDQ, mean ± sd	4.6 ± 1.7	4.7 ± 1.7	4.5 ± 1.7	0.55
- A	1.38 ± 0.6	1.41 ± 0.6	1.31 ± 0.6	0.45
- B	1.38 ± 0.6	1.43 ± 0.6	1.31 ± 0.6	0.51
- DR	1.02 ± 0.7	0.98 ± 0.7	1.05 ± 0.7	0.62
- DQ	0.84 ± 0.7	0.83 ± 0.7	0.87 ± 0.7	0.87
Total molecular mismatch, mean ± sd	45.8 ± 19.5	48.6 ± 20.4	42.4 ± 17.5	0.27
-AB	27 ± 10.6	28 ± 10.4	24.5 ± 10.3	0.08
-DR	12.6 ± 10	12.8 ± 10.9	12.0 ± 8.2	0.84
-DQ	7.2 ± 5.9	7.8 ± 6.2	6.8 ± 5.5	0.43
Viral infection during KT, *N* (%)	14 (15.6%)	8 (16.7%)	6 (14.3%)	0.76
Noncompliance, *N* (%)	16 (17.8%)	9 (18.8%)	7 (16.7%)	0.79
Acute rejection during KT, *N* (%)	38 (42.2%)	19 (39.6%)	19 (45.2%)	0.59
Graft duration (months), median [IQR]	115 [60–178]	103 [50–157]	120 [78–238]	0.06
Cause of graft failure, *N* (%)				0.53
Immunological	41 (45.6%)	21 (43.8%)	19 (45.2%)	
IFTA	39 (43.3%)	20 (41.7%)	19 (45.2%)	
Other	10 (11.1%)	3 (7.1%)	7 (14.6%)	
Hemodialysis, *N* (%)	81 (90.0%)	44 (91.7%)	37 (88.1%)	0.57
CVC-T, *N* (%)	22 (24.4%)	12 (25.0%)	10 (23.8%)	0.75
Transfusions during FU, *N* (%)	19 (21.1%)	12 (25.0%)	7 (16.7%)	0.33

cPRA: calculated panel reactive antibodies; DSA: donor specific antibodies; KT: kidney transplant; IS: immunosuppression; CNI: calcineurin inhibitor; mTOR: mammalian target of rapamycin inhibitor; IFTA: interstitial fibrosis and tubular atrophy; CVC-T: tunneled central venous catheter; FU - follow-up.

The rates of acute rejection, IS noncompliance, or viral infections (CMV or BK virus) in the failed graft were similar between groups. The mean kidney allograft survival was 133.8 ± 92 months, with chronic allograft nephropathy and interstitial fibrosis with tubular atrophy (IF/TA) being the most common cause of graft loss. No pregnancies were registered during the follow-up, and the blood transfusion rate was similar between the groups.

### Impact of CNI withdrawal on HLA sensitization and GIS

**Figure 2.**
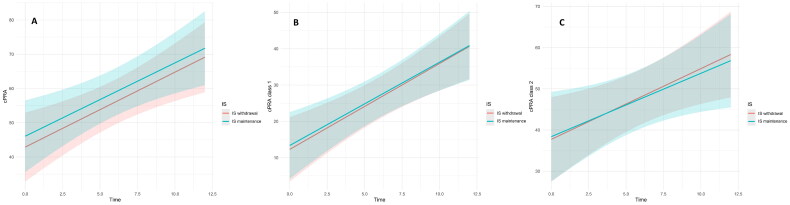
Variation in Total cPRA (A), cPRA class I (B), and cPRA class II (C), for rapid withdrawal and prolong CNI groups, as predicted by the fitted mixed model. Solid lines represent the predicted cPRA level at the time point in the x-axis, and the shaded areas are 95% confidence intervals.

At the time of allograft failure, the median cPRA was 47.5% [2–84], primarily due to class II sensitization. At T12 post-allograft failure, the mean cPRA increased to 85% [43–98], with 21 patients (23%) becoming highly sensitized. There were no significant differences in cPRA levels between the groups at T0, T6, or T12. (Supplementary Material - Table S1). A linear mixed model indicated an average monthly increase in cPRA of 2% (coefficient = 2.033, *p* < 0.001) following graft loss. The growth rate in cPRA over the first year did not differ between the IS groups for either class I or class II HLA alloantigens (*p* = 0.800) **(**Figure 2(A–C)**).** Additionally, we found no differences in the rate of cPRA increase when using an MFI cutoff of 2000 (Supplementary Material Figure S1). Analyzing cPRA as a categorical variable also revealed no differences between groups in the rate of patients progressing to a higher sensitization category at T12 **(**Table 2**),** independently of the cPRA category at T0 (Supplementary Material Figure S2(A–C)). At T12, the development of dnDSA at T6 and T12 did not differ between the groups (Table 2 and Supplementary Material Table S2).

**Table 2.** Increase in HLA sensitization and graft intolerance syndrome at T12 postgraft failure between is groups.

	Rapid Withdrawal	Prolonged CNI	*p*
Increase in cPRA T0 to T12, *N* (%)	21/40 (52.5%)	21/38(55.3%)	0.82
De novo DSA T0 to T12, *N* (%)	22/45(48.9%)	20/39(51.3%)	0.9
**GIS at T12,*****N*** (%)	**11/48(22.9%)**	**2/42(4.8%)**	**0.017**
Composite outcome at T12*, *N* (%)	25/40(62.5%)	25/36(69.4%)	0.63

CNI: calcineurin inhibitor; PRA: panel reactive antibody; DSA: donor-specific antibody.

*The composite outcome was defined as an increase in the cPRA category and/or *de novo* donor-specific antibodies and/or graft intolerance syndrome at T12.

**Figure 3.**
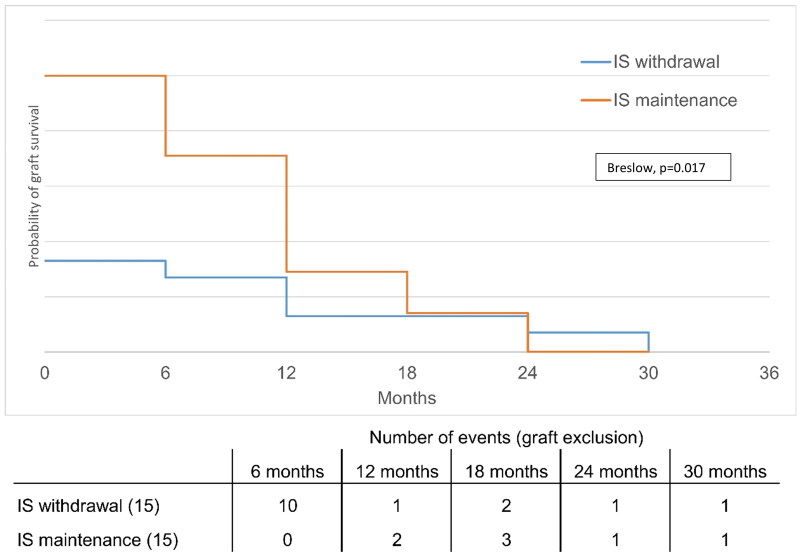
Survival analysis for graft removal according to immunosuppression post-graft failure group. IS – immunosuppression.

GIS occurred in 22 patients (24.4%) during follow-up, at a median time post-graft failure of 258 [132-479] days. For patients with prolonged CNI withdrawal, GIS occurred significantly later (Figure 3). First-year GIS occurred in 13 patients (13/22, 59%), and all patients needed graft removal at a median time of 143 days [115-201]. Graft removal was performed *via* transplant nephrectomy in eight patients (61.5%) and endovascular embolization in five patients (38.5%). Patients who prolonged CNI for at least 6 months had significantly lower graft removal rates (4.8% vs. 23%, *p* = 0.015) **(**Table 2**)**. Multivariate analysis adjusted for graft duration, HLA mismatch, acute rejection, noncompliance, and IS withdrawal showed that previous acute rejection and rapid CNI withdrawal were independent risk factors for first-year GIS **(**Table 3**)**.

**Table 3.** Multivariate analysis of the risk factors for first-year graft removal due to graft intolerance syndrome.

	OR [95% CI]	*p*
Rapid CNI withdrawal	**5.9 [1.2–32.1]**	**0.039**
Graft survival	0.99 [0.98–1.004]	0.21
Acute rejection	**5.6 [1.1–27.8]**	**0.036**
Noncompliance	2.3 [0.06–2.96]	0.39
Allelic mismatch	1.01 [0.97–1.04]	0.72

CNI: calcineurin inhibitors.

We found no differences between IS withdrawal strategy groups in the composite outcome of increased cPRA category, dnDSA, and GIS incidence at T12 **(**Table 2**)**.

### Risk factors for increased cPRA category at T12

Considering the high sensitization rate at T12, we analyzed the risk factors for increased cPRA category one year after graft loss. After excluding four highly sensitized patients at T0, a multivariate analysis adjusted for age, number of mismatches, acute rejection during KT, and allograft nephrectomy was performed. Graft removal within the first-year post-graft failure (OR 10.9 [1.1-112.5]) and the number of HLA mismatches were significantly associated with a higher likelihood of an increased cPRA category at T12 **(**Table 4**).** Each allelic HLA mismatch raised the risk by 1.7 times, while each eplet mismatch increased the risk by 1.04. Given that the molecular mismatch magnitude is about 10 times that of allelic mismatch (4.6 ± 1.7 vs. 45.8 ± 19.5, Table 1), every 10 eplet mismatches raised the risk of a cPRA category increase by 10.4 times. Only class II mismatches significantly affected sensitization when evaluating HLA classes separately.

**Table 4.** Multivariate analyses evaluating risk factors for increasing cPRA category at T12 post graft failure.

	Model 1		Model 2		Model 3	
Variable	OR [95%, CI]	*p*	OR [95%, CI]	*p*	OR [95%, CI]	*p*
Age at graft loss	0.96 [0.9–1.1]	0.21	0.96 [0.9–1.02]	0.2	0.94 [0.89–1.0]	0.052
Total allelic mismatch	**1.7 [1.2–2.5]**	**0.004**				
Class I allelic mismatch			1.27 [0.7–2.4]	0.44		
Class II allelic mismatch			**3.7 [1.5–9.3]**	**0.004**		
Total eplet mistmatch					**1.04 [1.01–1.07]**	**0.014**
Acute rejection	2.5 [0.8–8.4]	0.118	2.05 [0.6–6.8]	0.24	1.94 [0.62–6.0]	0.25
Graft removal	**10.9 [1.1–112.5]**	**0.04**	**11 [1.1–122]**	**0.048**	**10.6 [1.1–103]**	**0.041**

OR: odds ratio; CI: confidence interval.

Model 1: Total allelic mismatch (R^2^_Naglekkerke_=0.353); Model 2: Class I and II allelic mismatch (R^2^_Naglekkerke_=0.34); Model 3: Total eplet mismatch (R^2^_Naglekkerke_=0.33).

### Impact of CNI withdrawal on residual diuresis, hospitalization, and mortality

Patients who underwent prolonged CNI therapy exhibited a higher RKF at six months after allograft failure (800 [200–1,000] mL vs. 200 [0–500] mL, *p* = 0.001) (Table 5). In the first year following graft failure, there were 20 recorded hospitalizations, primarily due to GIS-related graft removal (*N* = 11) and all-cause infections (*N* = 6). Patients in the Rapid Withdrawal Group experienced a higher overall rate of hospitalizations (31% vs. 12%, *p* = 0.036) and had earlier hospitalizations in the post-graft failure period. At follow-up, three infection-related hospitalizations in the Prolonging CNI Group were registered: two patients due to pyelonephritis and one patient with a respiratory infection, with a mean hospitalization time of 6 ± 2.1 days. No differences were noted between the groups regarding retransplantation rates or all-cause mortality. (Table 5**)**.

**Table 5.** Clinical outcomes post graft failure between groups.

	Rapid withdrawal *N* = 48	Prolonged CNI *N* = 42	
Residual diuresis 6 months post graft failure (mL), median [IQR]	200 [0-500]	800 [200-1,000]	**0.001**
Hospital admission 1^st^ year, *N* (%)	15 (31.3%)	5 (11.9%)	**0.036**
Time to admission, median [IQR]	134 [115–189]	220 [81–220]	0.6
Cause of admission, N(%)			0.35
Graft intolerant syndrome	9 (60.0%)	2 (40.0%)	0.44
Infectious	3 (20.0%)	3 (60.0%)	0.091
Cardiovascular	2 (13.3%)	0 (0%)	
Neoplasia	1 (6.7%)	0 (0%)	
Retransplantation	2 (4.0%)	2 (4.8%)	0.86
Time to retransplant (months), median [IQR]	29.5 [26–33]	34.5 [30–39]	0.84
Death, *N*(%)	4% (2)	4.8% (2)	0.86
Infectious		1 (50%)	
Carviovascular		1 (50%)	
Unknown	2 (100%)		
Time to death (days), median [IQR]	912 [551–1273]	790 [468–1113]	0.9

IQR – interquartile range.

## Discussion

In our cohort, prolonging IS with CNI for six months did not prevent HLA sensitization, defined by an increase in cPRA or the development of dnDSA, during the first year after graft failure. Our findings align with a previous prospective study of IS withdrawal after graft failure. This study, conducted across 16 Canadian centers, found that continuing IS treatment with more than one drug after graft loss did not significantly change the cPRA levels [27]. Similarly, the first published systemic review and meta-analysis concluded that extending IS after allograft failure did not impact HLA sensitization or major clinical outcomes compared with rapid withdrawal [29].

In contrast, several retrospective studies have suggested that extending the use of CNIs for 3–6 months after graft loss is associated with lower rates of HLA sensitization. The first studies evaluating the impact of maintaining IS after graft failure on HLA sensitization have reported that rapid withdrawal of IS was an independent risk factor for increased HLA sensitization. However, these studies measured HLA sensitization using cytotoxic PRA, which may have underestimated the accurate sensitization rates. Additionally, many of these studies included multi-organ transplant patients who were maintained on high levels of IS to preserve the function of other organs [25,30,31].

Recent studies that used solid-phase assays have demonstrated that maintaining a combination of steroids, CNIs, and/or antiproliferative agents for three to six months following graft failure reduces the risk of HLA sensitization compared to a steroid-only approach [20,21,26,32]. This advantage may be related to the continued use of triple IS in several patients, which was not observed in our cohort. However, current recommendations indicate that antiproliferative drugs should be discontinued at the time of graft failure unless re-transplantation is anticipated within a short timeframe [33,34]. Other authors have reported a decrease in dnDSA or prevention of a cPRA increase by maintaining more extended periods of CNI. However, the control groups completely withdrew from IS, including steroids, making it more challenging to contextualize the results [19,23,24]. A recent paper from Allesina et al. showed that patients in a late-withdrawal group presented significantly lower cPRA and very low rates of highly sensitized patients at 12- and 24-month postgraft failure compared to rapid IS withdrawal. CNI-based IS was maintained for a median of 1.8 years, with medium tacrolimus and cyclosporin levels of 3.14 and 45.8 ng/mL [35]. Several differences might explain the different results from our cohort. We have a shorter duration of CNI-based IS (6 months versus 22 months), and our cohort had a significantly higher cPRA at graft loss (47.5% vs 0%), which makes prevention of further HLA sensitization with low doses of CNI harder to achieve [36].

In our cohort, the mean CNI trough levels measured at T6, 3.07 ± 0.7 ng/dL for tacrolimus and 41 ± 18.5 ng/dL for cyclosporine, which is significantly lower than those recommended for a functioning graft [37]. Lucisano et al. have also demonstrated that lower tacrolimus levels following allograft failure were an independent risk factor for DSA development 24 months after graft loss [18]. However, recommendations concerning the IS target doses and levels of post-graft loss are scarce.

An important finding of our study was that prolonging IS significantly reduced the rate of GIS, which is associated with significant morbidity [38] and was the leading cause of hospitalization post-graft failure in our cohort. Furthermore, first-year graft removal was associated with an 11-fold increase in sensitization risk. Martin et al. have published similar results of a protective effect of prolonging CNI against GIS despite no influence on the risk of increased HLA sensitization. A possible explanation might be that CNI maintenance primarily affects T-cell mediated GIS, while its effect on B-cell mediated sensitization is less direct [39,40]. In patients with a retained failed graft, a "sponge effect" could also occur, where DSAs bind to the residual graft instead of circulating in the blood, making it appear as though there is no significant difference in PRA or DSA between groups [18,22]. The study’s finding that sensitization was significantly higher in the nephrectomy group supports this explanation.

An important finding from our cohort is the low prevalence of infection-related hospitalization or death in the Prolonged CNI Group. The risk of mortality is exceptionally high in the initial months following graft failure, and infection resulting from prolonged exposure to IS is often viewed as a significant risk factor [34]. In the absence of another functioning graft, prolonging IS post-graft failure represents a difficult decision for the participating nephrologists. However, the most recent published data has shown similar infection rates in the prolonged IS group to those of the rapid withdrawal group, which aligns with our cohort results [20,23,26,27,35,41].

Portugal has one of the highest rates of highly sensitized patients waitlisted for KT (approximately 35%), with the majority waiting for subsequent kidney grafts [15]. In our cohort, the mean cPRA at T12 was 85%, regardless of the IS withdrawal group, which significantly impacts the prospects of retransplantation. This highlights the importance of identifying factors that influence the increase in cPRA after allograft failure. In addition to GIS, we found that the number of HLA mismatches, both allelic and molecular, was a significant risk factor for sensitization at T12, independent of IS withdrawal. Due to the high homology between different HLA antigens, each mismatch can produce multiple HLA antibodies, exponentially increasing cPRA [5]. Mismatches at class 2 loci, particularly the -DQ locus, are becoming increasingly important, and some organ allocation systems now prioritize class II matching [42,43]. Our findings support this approach, as we observed that class II mismatches were the most substantial contributors to sensitization and that most patients were sensitized to class II antigens at T0, T6, and T12. Molecular mismatches were strongly associated with the degree of sensitization post-graft failure, as previously reported [42].

In conclusion, a slower CNI withdrawal strategy did not prevent a composite outcome of increased cPRA, dnDSA, and GIS, which we believe is linked to low CNI exposure. However, prolonging CNI effectively prevented GIS and was associated with better RKF post-graft failure without increasing the risk of all-cause hospitalization or mortality. For patients with kidney graft failure who have prospects for retransplantation and do not have severe complications or side effects from CNI, a slower CNI withdrawal strategy should be considered. Patients at a high risk of GIS, including those with a history of acute rejection during transplantation and those with a high HLA mismatch, benefit from slower tapering of CNI to decrease the need for graft nephrectomy. An important conclusion of our cohort is the necessity of providing the best HLA match, particularly for class II antigens, since HLA mismatch strongly predicted T12 sensitization regardless of the CNI withdrawal scheme. Tools such as PIRCHE can help optimize donor-recipient compatibility, contribute to graft longevity, and reduce HLA sensitization post-graft failure [44].

The strengths of this study include its prospective, quasi-randomized design, which allows for a comparison between two demographically and clinically similar groups **(**Table 1**)** and a thorough and rigorous follow-up of HLA sensitization. The study also benefited from homogeneous IS withdrawal strategies, careful monitoring of CNI dosing, and solid-phase and molecular assays. However, this study has some limitations: it was conducted at a single center, had a small sample size, and had missing values for HLA sensitization at T6, which weakened its statistical power. More prospective studies with larger, multicenter cohorts are needed to understand which patients might benefit from rapid or slower IS withdrawal. This will help move toward precision medicine for the management of graft failure.

## Supplementary Material

Supplemental Material

Supplemental Material
