# Supplementary material for: Prolonging calcineurin inhibitor therapy post kidney allograft failure: a prospective study
Source: Ren Fail. 2025 Mar 30;47(1):2483386. doi: 10.1080/0886022X.2025.2483386 (PMC11960309; doi:10.1080/0886022X.2025.2483386)
Supplement: Supplemental Material [file IRNF_A_2483386_SM4616.docx]

**SUPPLEMENTARY MATERIAL**

**Tables**

Table S1 – cPRA values between groups at T0, T6, and T12 post-graft failure.

|  | Total | Rapid  Withdrawal | Prolonged CNI | p |
| --- | --- | --- | --- | --- |
| **cPRA t0 (N=90),**  **median [IQR]** | 47.5 [2-84] | 44.7 [1-96.8] | 45.3[0-84.2] | 0.77 |
| Class I | 0 [0-20] | 0 [0-13] | 0.5 [0-20] | 0.20 |
| Class II | 31.9 [0-81] | 40 [0-64.6] | 30.5[0-81] | 0.79 |
| **cPRA T6 (N=67),**  **median [IQR]** | 68.1 [7.6-97] | 61.9 [3.3-96.3] | 66.3 [24-99.6] | 0.95 |
| Class I | 0 [0-55.7] | 7 [0-58] | 0 [0-48.2] | 0.31 |
| Class II | 47.4 [0-85.8] | 48.7 [0-85.6] | 39 [0-81.2] | 0.67 |
| **cPRA T12 (N=82),**  **median [IQR]** | 85 [43-98] | 91.1 [45-98] | 89.3 [48-98] | 0.85 |
| Class I | 21 [0-81] | 30.3 [0-83] | 26.9 [0-89] | 0.55 |
| Class II | 65.7 [0-92.6] | 76.5 [17.2-93] | 78 [0-95] | 0.87 |

CNI –calcineurin inhibitor. The results are expressed as median [interquartile ranges]

Table S2 – Donor-specific antibodies at T0 and the rate of de novo donor-specific antibodies between groups at different time points.

|  | Rapid Withdrawal | Prolonged CNI | p |
| --- | --- | --- | --- |
| **DSA_T0 (N=90), N (%)** | 20 (41.7%) | 17 (40.5%) | 0.93 |
| Class I | 9 (18.8%) | 9 (21.4%) | 0.75 |
| Class II | 20 (41.7%) | 17 (40.5%) | 0.90 |
| ***De novo* DSA T0 to T6 (N=66), N (%)** | 11 (32.4%) | 11 (34.4%) | 0.86 |
| Class I | 7 (19.4%) | 5 (15.6%) | 0.68 |
| Class II | 8 (23.5%) | 9 (28.1%) | 0.67 |
| ***De novo* DSA T0 to T12 (N=84), N (%)** | 22 (48.9%) | 20 (51.3%) | 0.82 |
| Class I | 16 (35.6%) | 12 (30.8%) | 0.64 |
| Class II | 21 (46.7%) | 20 (51.3%) | 0.67 |
| ***De novo* DSA T6 to T12 (N=67), N (%)** | 10 (28.6%) | 7 (21.9%) | 0.53 |
| Class I | 5 (14.3%) | 2 (6.3%) | 0.28 |
| Class II | 7 (21.2%) | 5 (18.8%) | 0.80 |

CNI- calcineurin inhibitor; DSA – donor specific antibodies;
