# Supplementary material for: Prolonging calcineurin inhibitor therapy post kidney allograft failure: a prospective study
Source: Ren Fail. 2025 Mar 30;47(1):2483386. doi: 10.1080/0886022X.2025.2483386 (PMC11960309; doi:10.1080/0886022X.2025.2483386)
Supplement: Supplemental Material [file IRNF_A_2483386_SM4613.pptx]

## Slide 1
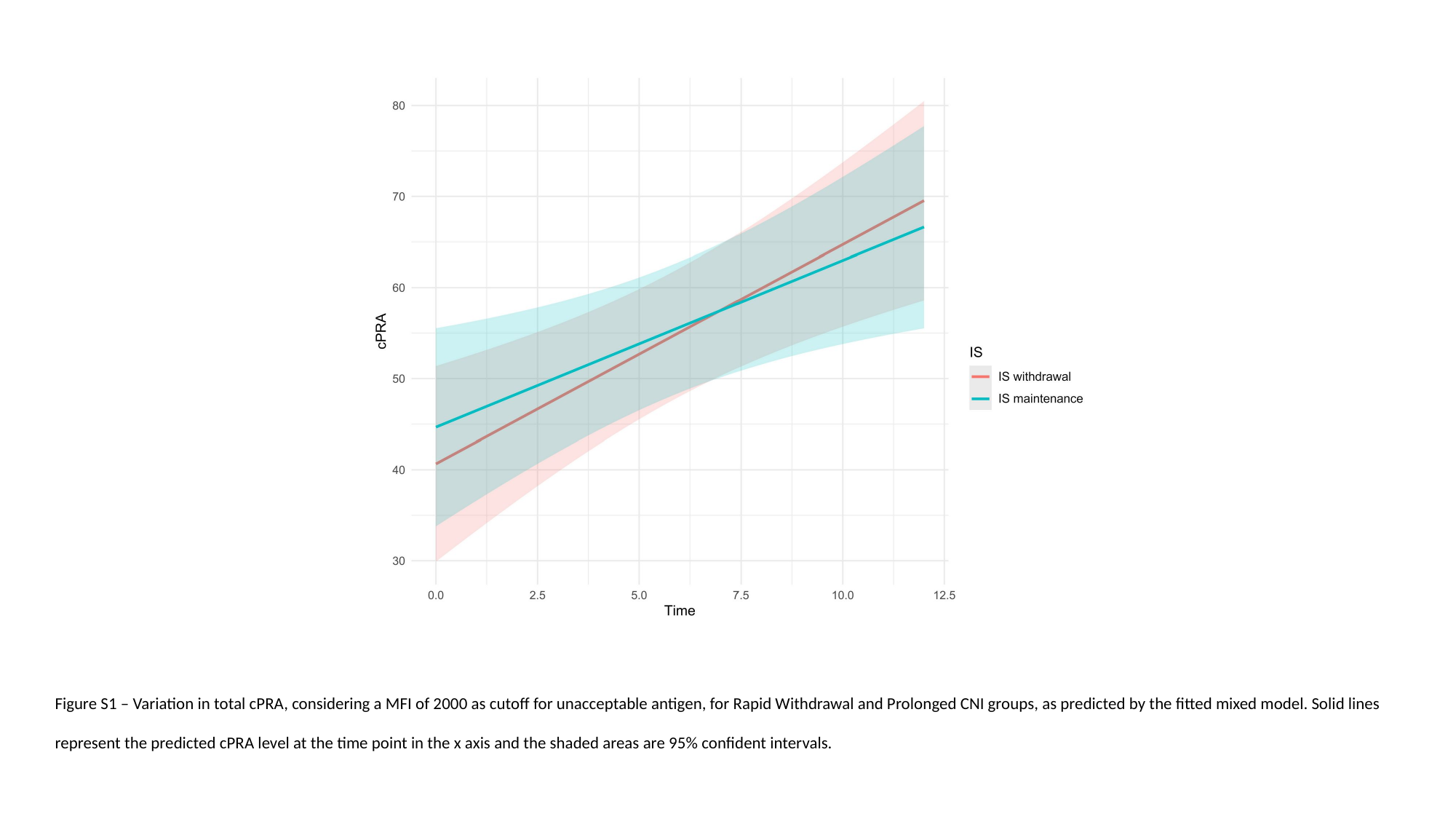

Figure S1 – Variation in total cPRA, considering a MFI of 2000 as cutoff for unacceptable antigen, for Rapid Withdrawal and Prolonged CNI groups, as predicted by the fitted mixed model. Solid lines represent the predicted cPRA level at the time point in the x axis and the shaded areas are 95% confident intervals.

## Slide 2
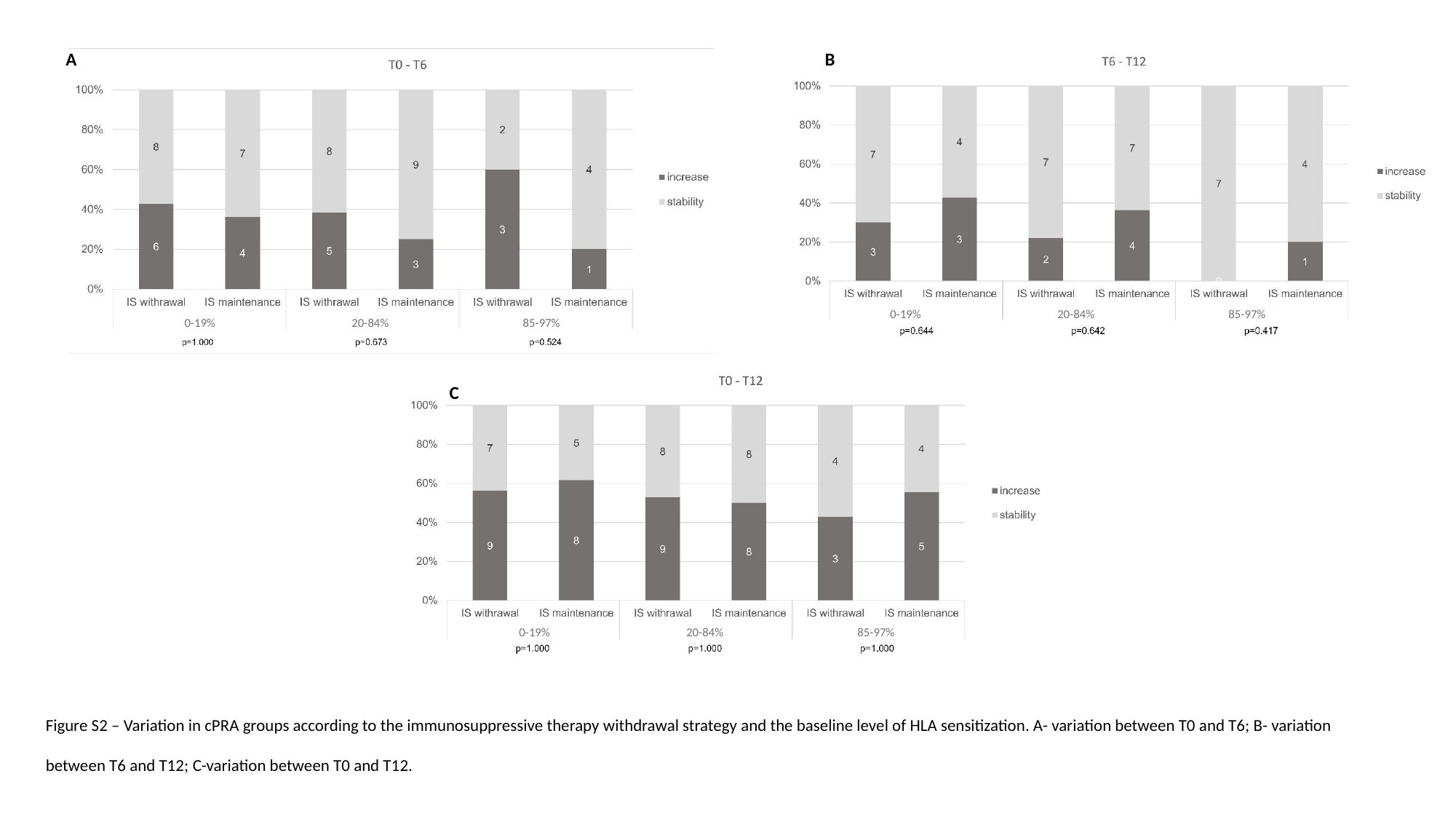

A
B
0-19%
20-84%
85-97%
0-19%
20-84%
85-97%
C
0-19%
20-84%
85-97%
Figure S2 – Variation in cPRA groups according to the immunosuppressive therapy withdrawal strategy and the baseline level of HLA sensitization. A- variation between T0 and T6; B- variation between T6 and T12; C-variation between T0 and T12.
